# Computational frameworks for enhanced extracellular vesicle biomarker discovery

**DOI:** 10.1038/s12276-025-01622-x

**Published:** 2026-01-14

**Authors:** Jina Kim, Ju Dong Yang, Vatche G. Agopian, Yazhen Zhu, Hsian-Rong Tseng, Sungyong You

**Affiliations:** 1 Department of Urology, Cedars-Sinai Medical Center, Los Angeles, CA USA; 2 Department of Computational Biomedicine, Cedars-Sinai Medical Center, Los Angeles, CA USA; 3 Samuel Oschin Comprehensive Cancer Institute, Cedars-Sinai Medical Center, Los Angeles, CA USA; 4 Karsh Division of Gastroenterology and Hepatology, Cedars-Sinai Medical Center, Los Angeles, CA USA; 5 Comprehensive Transplant Center, Cedars-Sinai Medical Center, Los Angeles, CA USA; 6 Department of Surgery, David Geffen School of Medicine, University of California, Los Angeles, Los Angeles, CA USA; 7 Jonsson Comprehensive Cancer Center, University of California, Los Angeles, Los Angeles, CA USA; 8 Department of Pathology and Laboratory Medicine, David Geffen School of Medicine, University of California, Los Angeles, Los Angeles, CA USA; 9 Department of Molecular and Medical Pharmacology, California Nanosystems Institute, Crump Institute for Molecular Imaging, University of California, Los Angeles, Los Angeles, CA USA

**Keywords:** Computational models, Biomarkers

## Abstract

Extracellular vesicles (EVs) are emerging as promising noninvasive biomarkers, yet their clinical translation faces substantial hurdles, primarily due to the challenge of identifying assay-compatible markers. Here, in this Review, we outline sophisticated computational frameworks, particularly leveraging artificial intelligence, to bridge this gap. We detail the integration of diverse data resources, including disease-specific omics, EV, protein localization, tissue-specific, drug, model system and immune databases. This Review comprehensively describes computational selection strategies, from rule-based sequential filtering to advanced machine learning for data fusion and deep learning for multi-omics integration. Crucially, it discusses the refinement of biomarker candidates using artificial-intelligence-driven predictions of protein structure and physicochemical properties, ensuring compatibility with existing assay systems. By systematically evaluating biomarkers for predictive performance, biological plausibility and clinical utility, this framework aims to accelerate the transition of EV research from discovery to clinical application, thereby enhancing precision medicine.

## Bridging research and practice in extracellular vesicle biomarkers

Extracellular vesicles (EVs) are nanosized particles released by cells, carrying RNA, proteins and lipids. They hold promise as noninvasive markers for diseases such as cancer and neurodegenerative disorders. However, using EVs in clinical settings is challenging. Many candidate markers identified in research do not work well with current testing methods. In 2021 alone, over 1,000 studies on EV markers were published, but only 4 were clinically validated. This Review emphasizes the need for advanced computational tools to identify clinically viable markers. The authors discuss various data resources and computational strategies, including artificial intelligence approaches that predict protein structures, interactions and assay compatibility to prioritize candidates. The study concludes that combining advanced computational approaches with EV assays can speed up the transition from research to clinical practice.

This summary was initially drafted using artificial intelligence, then revised and fact-checked by the author.

## Introduction

**Fig. 1:**
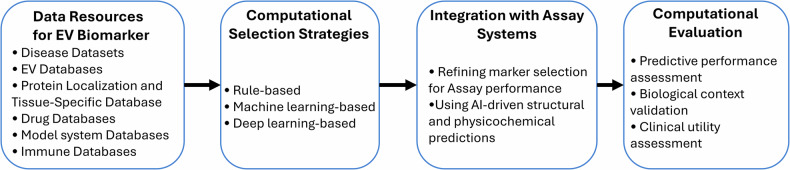
From data to clinical translation: a framework for EV biomarker discovery. This flowchart outlines the systematic workflow for EV biomarker identification.

Extracellular vesicles (EVs) are nanosized vesicles secreted by cells, carrying several molecules such as RNAs, proteins, lipids and DNA^1,2^. Due to their molecular complexity and cell-type specificity, EVs have emerged as a promising source of noninvasive biomarkers for various diseases, including cancers^3–5^ and neurodegenerative disorders^6–8^. Over the past decade, advances in omics technologies and EV isolation methods have greatly expanded the landscape of potential EV-based biomarkers. Despite these advances, translating EV biomarkers into clinical practice remains challenging^9^. Conventional discovery strategies have primarily focused on identifying disease-associated molecules through differential expression or multi-omics integration. However, markers identified solely on the basis of disease relevance may not always be compatible with available assay platforms owing to molecular properties^10–12^. A number of promising candidates uncovered by the conventional methodologies lack the necessary accessibility for antibody binding in assays or the structural stability required for consistent measurement, thereby hindering their transition from discovery to clinical utility. For instance, in 2021 alone, there were more than 1000 research papers published on EV-based biomarkers, yet only 4 EV-based biomarker assays have been clinically validated^13,14^. Therefore, identifying assay-eligible biomarkers is increasingly recognized as a crucial step toward clinical utility. These challenges highlight the need for developing sophisticated computational frameworks enabling precise biomarker identification for clinical use^15^. In response to this need, recent breakthroughs in artificial intelligence (AI), particularly in protein structure prediction and molecular interaction modeling^16^, have introduced novel avenues for biomedical research^17–19^. These technologies may offer unprecedented opportunities to bridge this gap between computational EV marker discovery and clinical assay design by prioritizing markers based not only on biological relevance but also on molecular characteristics and interactions. In this Review, we comprehensively introduce the computational frameworks for the identification of candidate EV biomarkers. Here, we first summarize several data resources that are important for the discovery of EV biomarkers from a computational perspective, including specialized disease-specific omics databases, EV databases, protein localization and tissue-specific databases, drug databases, model system databases and immune databases, highlighting their distinct applications. In the following section, we will outline a variety of computational selection strategies, ranging from stepwise filtering methods based on sequential selection to advanced AI techniques for identifying informative EV biomarkers. Furthermore, the critical aspect of integrating computational insights with advanced assay systems is discussed, emphasizing how structural and physicochemical predictions based on AI can refine EV biomarker candidates for the application of the assay. In conclusion, this Review summarizes future perspectives and challenges in this field. Harnessing the synergistic potential of cutting-edge computational techniques in conjunction with EV assays can accelerate the transition of EV research from promising research findings into routine clinical practice (Fig. 1).

## Data resources and their potential utilities

Identifying EV biomarkers for its successful clinical application is complex, necessitating integration of large heterogeneous biological and clinical datasets. As EV research rapidly expands, so does the quantity and diversity of available resources, spanning from the molecular information of EVs to comprehensive disease profiles, protein location, drug interactions, model system characteristics and responses in immune cells. Systematic knowledge and use of such data resources are essential to expedite the discovery of robust, assay-eligible EV biomarkers. This section discusses the major types of databases for EV biomarker discovery and the role each database type plays in marker selection strategies (Table 1).

**Table 1:** Data resources for EV biomarker discovery.

Data category	Database	Characteristic	Reference
Disease datasets	TCGA	TCGA systematically collected molecular and clinical data from over 11,000 patients with cancer across more than 20,000 tumor and normal samples. TCGA used diverse high-throughput sequencing data of DNA, RNA, methylation and protein expression and integrated clinical metadata	^62^
	CPTAC	Large-scale proteogenomic datasets by integrating DNA, RNA and protein measurements across cancer and adjacent normal tissues to investigate comprehensive protein quantity, uncover cancer subtypes and elucidate pathways through post-translational modifications	^63^
	TARGET	Focused on molecularly characterizing pediatric cancers through comprehensive genomic, transcriptomic and epigenomic landscapes to identify novel therapeutic targets and prognostic markers and improve treatment strategies	https://www.cancer.gov/ccg/research/genome-sequencing/target
	AD Knowledge Portal	Offering multi-omics datasets, including genomic variants and transcriptomic data from individuals with aging-related diseases such as Alzheimer’s	^64^
	COPD Cell Atlas	A public web tool designed to allow exploration of single-cell RNA sequencing data generated from the lungs of individuals diagnosed with COPD	^65^
	Prostate Cancer Transcriptome Atlas	A user-friendly web tool providing access to organized transcriptome data from 1321 prostate cancer clinical specimens, featuring clinically relevant subtype information, Gleason grade and metastasis status	^66^
EV databases	Vesiclepedia	50,000 RNA and 567,000 protein entries from 3500 EV studies; curated EV cargo compendium	^67^
	EVpedia	>172,000 vesicular components integrated from 6800+ publications (proteins, RNAs and lipids)	^68^
	ExoCarta	Manually curated database of exosomal proteins, RNAs and lipids (>41,000 proteins, >7500 RNAs); provides PPI networks and pathway annotations, and supports functional analysis via FunRich	^69^
	EV-TRACK	Crowdsourced database tracking EV study methods for standardization (>1200 studies)	^70^
	EV-COMM	1481 curated EV-mediated interaction records across intercellular and interspecies studies	^71^
Protein localization and tissue-specific databases	TCSA	Integrates genomic, functional and drug-response data to map cancer-specific surface proteins (GESPs). Uses a GESP score (combining multiple evidence sources) to prioritize actionable targets for drug and biomarker development	^72^
	HPA	Maps tissue and subcellular localization of human proteins and RNAs, including identification of secreted proteins	^73,74^
	GTEx	Links genetic variants to gene expression across diverse normal human tissues	^75^
Drug databases	ClinicalTrials	Tracks biomarkers under clinical investigation, trials or diagnostic monitoring	https://clinicaltrials.gov/
	Pharos	Categorizes targets by druggability (Tclin/Tchem/Tbio/Tdark) and links to ligands/diseases	^76^
	DrugBank	Drug, target, mechanism and interaction data for >11,800 compounds (US Food and Drug Administration-approved and investigational)	^77^
	Drugst.One	Visualizes protein–drug–disease networks for repositioning and prioritization	^78^
	NeDRex	Network-based platform for drug–disease module discovery; integrates a Cytoscape app and application programming interface	^79^
	LINCS	Perturbation-based transcriptomic and proteomic profiles for drugs and bioactive molecules	^80^
Model system databases	CCLE, DepMap	Providing deep molecular and pharmacologic profiling for nearly 1000 human cancer cell lines across ~36 lineages and functional genomics (CRISPR, RNAi) for gene dependency mapping	^81–83^
	PDMR	Biobank of patient-derived xenografts, organoids and fibroblasts; molecular and clinical metadata for preclinical testing	https://pdmr.cancer.gov/
Immune databases	DMAP	Gene expression profiles across human hematopoietic development stages.	^84^
	ImmGen	Transcriptomes of 230+ mouse immune cell populations, aiding immune marker exclusion	^85^
	ImmPort	Repository of >1000 immunology datasets with standardized metadata and programmatic access	^86^

(1) Disease datasets. Large-scale disease cohort datasets (for example, publicly available; The Cancer Genome Atlas (TCGA), CPTAC, TARGET, AD Knowledge Portal, COPD Cell Atlas, Prostate Cancer Transcriptome Atlas and so on) are crucial in identifying key disease-associated molecules that can serve as candidate EV biomarkers. These datasets are invaluable in the candidate generation stage, enabling identification of genes or proteins that are disease specific^20–22^, and can be used to validate detected EV biomarkers. For example, TCGA analysis was used to elucidate the role of their marker in tumors, which revealed its consistent overexpression across multiple cancers compared with normal tissues^23,24^.

(2) EV databases. EV databases such as Vesiclepedia, EVpedia, ExoCarta, EV-TRACK and EV-COMM systematically document the molecular contents (proteins, RNAs and lipids) of EVs and methodological details from thousands of EV studies. These resources are essential during the initial selection stage, where they enhance confidence in recurrent EV markers, support validation of isolation and extraction methods by enabling cross-comparison and help reconcile discrepancies across studies. For instance, colorectal cancer study and non-small-cell lung cancer study confirmed detected proteins as EV-related by matching them to these databases^23,25^, while ovarian cancer studies used them to filter out common EV proteins and prioritize lineage-specific EV biomarkers^26^.

(3) Protein localization and tissue-specific databases. Protein localization and tissue-specific databases (TCSA, HPA and GTEx) are supportive of EV biomarker discovery. These resources facilitate the refinement of biomarker candidates by identifying molecules with a high likelihood of being localized on EV membranes or within EVs, while also assessing their tissue specificity, thereby supporting the selection of optimal targets for the intended assay^20,27–29^. For example, the HPA was utilized for the initial identification of 488 proteins exclusively expressed in the brain, contributing to the validation of brain-specific EV biomarker candidates such as APLP1. TCSA was used to screen for surface proteins on pancreatic ductal adenocarcinoma EVs.

(4) Drug databases. Drug databases collectively enable researchers to enhance the translational potential of EV biomarker discovery. Pharos, DrugBank, ClinicalTrials.gov, Drugst.One, NeDRex and LINCS collectively provide data on drug-target relationships, ligand interactions, druggability tiers and ongoing clinical investigations. These databases can play a pivotal role in the target validation stage, allowing researchers to select EV biomarkers with therapeutic relevance or existing clinical interest. By filtering EV markers on the basis of therapeutic relevance with known drug associations, these resources help focus on targets more likely to be clinically actionable^27^.

(5) Model system databases. Only one tissue-based study cannot discover EV markers associated with the systemic responses to cancer. Databases of model organisms and cell lines are invaluable to explore and validate EV biomarkers experimentally. Resources such as the Cancer Cell Line Encyclopedia (CCLE), DepMap and the NCI’s Patient-Derived Models Repository (PDMR) provide comprehensive molecular characterization and pharmacological profiling of cell lines, xenografts and organoids. These resources provide crucial experimental contexts for preclinical selection based on the expression of identified EV biomarkers, or for discovering novel biomarkers across various tumor cell lines and organoid models^30–32^.

(6) Immune databases. Effective EV protein biomarker selection requires consideration of immune cell expression profiles to maintain disease specificity. DMAP, ImmGen and ImmPort curate gene expression profiles across human and mouse immune cells. Immune expression databases can facilitate the removal of immune-associated markers during the marker identification step, ensuring that selected biomarkers provide clearer, disease-specific information^20,22,27^. In addition, their immune information can provide an opportunity to identify essential EV biomarkers for predicting responses to immunotherapy in precision immuno-oncology^4^

## Selection strategies

EV biomarker identification involves considerable analytical challenges due to multi-omics and diverse data types from various experimental platforms and methodologies. Identification of clinically and biologically useful EV biomarkers in these datasets requires advanced computational feature selection strategies. The following section provides a comprehensive overview of these computational selection strategies, highlighting their strengths and limitations.

**Fig. 2:**
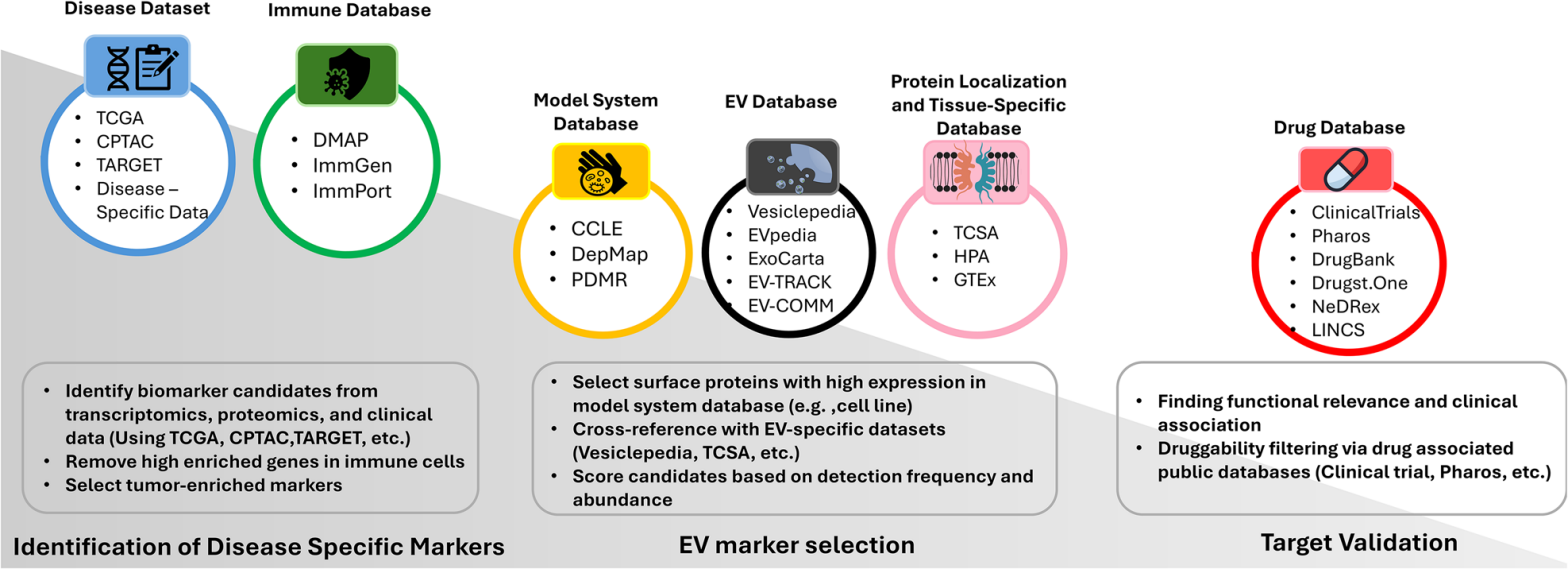
Generalized rule-based sequential selection using various databases. This figure illustrates a step-by-step filtering strategy to identify disease-specific EV markers.

(1) Rule-based sequential selection. Rule-based sequential selection for EV biomarker discovery involves a stepwise filtering framework that systematically integrates biological knowledge and multi-omics data to identify disease-specific EV markers. The process involves identifying disease-specific markers from disease datasets while removing broadly expressed ones referring to housekeeping or immune-related genes, then verifying their association and expression with model system databases, EV databases and molecular localization databases, as appropriate for the assay method. Finally, it prioritizes targets on the basis of their functional and therapeutic relevance to drug databases **(**Fig. 2).

This sequential selection serves as a representative example of a biologically driven, multilayered integration strategy for EV marker and could be adapted to different disease contexts. It further promotes reproducibility and robustness by reducing algorithmic bias and noise, while focusing on features that are consistently salient. In addition, selecting targets with druggability or clinical trial relevance enhances the translatability of the prioritized markers. However, this strategy has limitations. The effectiveness of this strategy relies on the availability and quality of public multi-omics data, which is not always the case, especially for rare or understudied conditions. Furthermore, the filtering criteria might exclude novel EV markers that have not yet been described in contemporary databases. Ultimately, computational prioritization alone cannot replace empirical validation, as functional assays are essential to confirm the diagnostic or therapeutic utility. This rule-based selection has been effectively applied in various cancer types to identify disease-specific EV markers. For instance, in pancreatic ductal adenocarcinoma, this sequential approach integrating CPTAC and CCLE proteomics with surface/EV association (TCSA, Vesiclepedia) led to the selection of MUC1, EGFR and TROP2, which were validated as diagnostic markers in patient plasma^27^. Similarly, researchers in osteosarcoma combined differential expression analysis with curated surfaceome lists and applied EV-related evidence to rank candidate EV surface proteins sequentially^20^. In brain-associated EV studies, the GTEx and HPA databases were utilized to evaluate brain specificity and select brain EV biomarkers across different datasets^28,33^. In hepatocellular carcinoma, this selection strategy involved identifying tumor-enriched genes while filtering highly expressed genes in immune cells using DMAP, followed by confirmation of vesicle association and therapeutic relevance^22^.

(2) Data fusion using machine learning (ML). ML methods have recently drawn attention as powerful tools to identify informative EV novel markers that may not be detectable through conventional statistical approaches. In this strategy, heterogeneous data (for example, mRNA, protein and clinical data) are fused into a ML model. ML algorithms process multi-omic profiles to implicitly weigh and select the most informative features, for example, for distinguishing disease from control samples (supervised learning) or for discovering underlying biology (unsupervised learning). ML-based data fusion can capture complex, nonlinear interactions between features. A primary advantage is to enable a comprehensive view and to discover biomarkers that show relatively weak contributions separately, while they play synergistically together. However, limitations remain, such as complexities in model building due to heterogeneous data types and the risk of overfitting in the absence of robust cross-validation strategies. Nevertheless, the integration of multi-omics data with ML has shown promising outcomes in disease-specific biomarker identification, especially when feature regularization or ensemble learning is used to reduce the risks of overfitting.

Several supervised ML algorithms have been widely adopted in EV biomarker discovery pipelines. Least Absolute Shrinkage and Selection Operator (LASSO) regression was applied to identify combinations of EV surface proteins (PLAU, ITGAX, ANXA1 and ITGA4) that distinguish Alzheimer’s disease from healthy controls^34^. Random forest, an ensemble method combining multiple decision trees, has been shown alongside LASSO regression and stepwise elimination to effectively rank feature importance and build models for pan-cancer detection of EV protein markers^35^. Support vector machines also excel at complex classification tasks by projecting data into higher-dimensional spaces using kernel functions to identify nonlinear decision boundaries. One study applied support vector machine modeling to plasma-derived EV proteomics data to build a seven-protein signature for early pancreatic cancer detection^36^. Beyond algorithms, integrating multiple ML approaches and data types is often essential due to the complexity of biological systems. Data Integration Analysis for Biomarker discovery using Latent cOmponents (DIABLO) maximizes correlation across multiple omics datasets while identifying key variables and disease phenotypes^37^. Similarly, AutoOmics provides an automated ML framework to select and optimize models across different omics layers before fusing latent features into final classifiers^38^, and iClusterBayes takes a Bayesian approach to integrative clustering, jointly modeling correlations across omics types to identify molecular subtypes in complex diseases^39^.

(3) Data integration using deep learning (DL). As biological datasets grow in complexity and size, AI is increasingly used to discover clinically relevant biomarkers. DL models (for example, convolutional neural networks, autoencoders and graph neural networks) can combine multimodal data and incorporate known biological networks. However, these models often function as black boxes, offering limited interpretability without additional explainability tools (for example, SHapley Additive exPlanations (SHAP), which is a unified explainable ML method). They also require large-scale datasets for training and involve complex model design and computational burden. Although still limited in EV biomarker research, DL tools leveraging multi-omics data for biomarker discovery are emerging, and this section reviews how DL models are being used in biomarker research. A major application lies in multi-omics data integration, where DL frameworks surpass conventional methods by capturing cross-modal relationships. For instance, MOGONET uses graph convolutional networks (GCNs) to integrate mRNA, microRNA (miRNA) and DNA methylation data, improving disease classification while identifying omics-specific biomarkers^40^. Similarly, GOAT, an attention-based graph neural network, combines multi-omics features with protein–protein interaction (PPI) networks to prioritize key regulatory factors, such as CTNNB1 and JUN, that were not detectable by traditional analysis^41^. DL approaches have proven effective in disease diagnosis and classification. In chronic obstructive pulmonary disease (COPD), a GCN-based framework was trained using gene expression and proteomic profiles onto a PPI network to distinguish diseased from normal samples, while explainable AI (xAI) techniques, including SHAP analysis, highlighted critical features such as CXCL11, IL-2 and CD48^42^. DL also supports identifying patterns and clustering data without labels, enabling the discovery of key features on clustering novel groups within disease multi-omics data, outlier detection and dimensionality reduction for biomarker discovery. Autoencoders, for example, can compress high-dimensional multi-omics data into lower-dimensional representations to identify subpopulations. Furthermore, important features can be identified by assessing which biomarkers most strongly influence the encoding through analyses such as SHAP or gradient-based attribution. A tool like Multi-omics Autoencoder Integration (MAUI) is based on a variational autoencoder (β-VAE). It integrates various data types (for example, gene expression, mutations and copy-number alterations) and stratifies colorectal cancer subtypes^43^. DeepProg combines autoencoders with ML to predict survival subtypes and classify high-risk groups using multi-omics data (for example, RNA sequencing, methylation and miRNA)^44^. Finally, CrossPred, a deep multi-encoder model, links exosomal miRNAs and intracellular mRNAs through a shared embedding space, effectively denoising data and identifying cancer-linked miRNAs and genes^45^. In scenarios where samples of labeled data are scarce, a DL model can leverage both labeled and unlabeled data to strengthen its power. DL can first learn general features from abundant unlabeled data, then refine these features using a small set of labeled samples. As an example, MOSEGCN integrates mRNA, miRNA and methylation data using GCNs and attention, propagating label information across graphs of patient samples with mixed labeled and unlabeled data. It demonstrated high accuracy (~83% for Alzheimer’s, ~87% for breast cancer subtypes) and identified meaningful disease-related genes^46^.

**Fig. 3:**
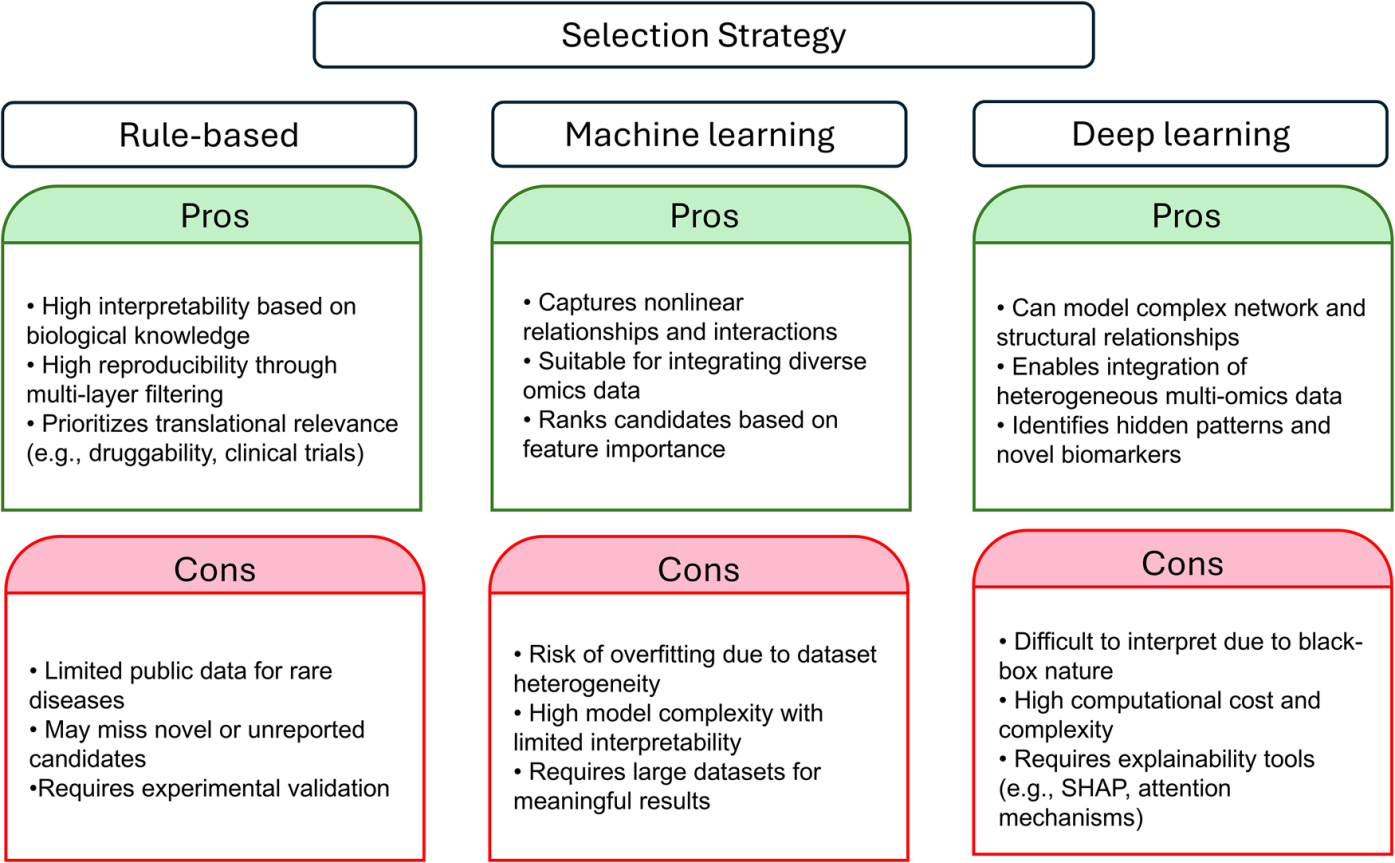
Comparison of selection strategies for EV biomarker identification. A comparative summary of Rule-based, Machine Learning, and Deep Learning approaches.

The most appropriate approach for EV biomarker discovery depends on the specific project’s needs. Rule-based sequential approaches are advantageous when working with well-annotated datasets that require clear filtering criteria, robust noise reduction and high interpretability, especially for rapidly prioritizing assay-compatible or clinically relevant markers. Conversely, ML is adept at capturing complex interactions and subtle signals in diverse multi-omics data, identifying biomarkers missed by rule-based strategies. However, it requires large datasets and is susceptible to bias induced by data noise. Emerging DL models can integrate heterogeneous multi-omics data and uncover hidden patterns in complex data. However, these models face challenges with interpretability, high computational burdens and complex design requirements. In addition, further extensive validation is needed as the application in the EV biomarker field is still limited (Fig. 3).

## Integration with assay system

For computationally identified EV biomarkers to reach clinical use, compatibility with existing assay platforms and reliable detection are crucial. After computational selection, translating EV biomarkers into clinical use requires ensuring compatibility with existing assay platforms. Many EV-derived RNAs and proteins exhibit structural or physicochemical traits that hinder detection. Prioritizing markers with assay-friendly properties is therefore critical. This section reviews existing EV assay systems for RNA and protein detection and then discusses how advanced computational insights can further refine biomarker selection for enhanced assay performance in terms of particularly structural and physicochemical predictions.

(1) Existing assay systems for RNA or protein detection. Bridging computational discovery and clinical use requires understanding currently available EV-based RNA and protein assays, which lead to noninvasive liquid biopsy technologies. One prominent example is the ExoDx Prostate (IntelliScore) test, a noninvasive urine test that utilizes EVs (exosomes) to assess a man’s risk of having high-grade prostate cancer. By analyzing specific RNA markers (PCA3, ERG and SPDEF) within these exosomes, the test provides a personalized risk score to help determine the necessity of an initial prostate biopsy, thereby potentially reducing unnecessary initial prostate biopsies^47,48^. Similarly, other examples include miR Sentinel test series analyzing small noncoding RNAs and miRNA patterns from urinary exosomes (for prostate and bladder cancer risk assessment)^49^, as well as the blood-based ClarityDx Prostate test (for high-grade prostate cancer protein analysis)^50^. Beyond these established tests, novel assays are continually emerging, such as an EV mRNA Digital Assay for hepatocellular carcinoma treatment response^22^, an EV Surface Protein Assay for noninvasive pancreatic cancer detection^27^ and an OS EV MMP Activity Assay for osteosarcoma monitoring, all showing substantial potential for early detection and disease management^20^.

(2) Computational strategies for biomarker refinement for enhanced EV detection assay system: protein structure and physicochemical properties. After initial biomarker selection, understanding molecular properties such as accessibility, binding efficiency and behavior in assay environments is critical for diagnostic or therapeutic use. Recent advances in AI have led to functional genomics models such as Evo2, AlphaGenome, LucaOne and ChatNT predicting genome function from DNA sequences and are driving progress across the bioindustry^51–54^. In line with these advancements, this section introduces AI tools that can be applied after biomarker selection, particularly those capable of predicting protein structures and modeling their interactions, which are essential for assay platform compatibility.

AlphaFold3 (AF3) is a notable advancement, capable of modeling diverse biomolecules (proteins, DNA and RNA) and their interactions at atomic resolution. Unlike traditional docking tools such as AutoDock Vina^55^, HADDOCK^56^, ClusPro^57^ or RosettaDock^58^, which require predetermined structures, AF3 can generate entire molecular complex structures end-to-end from sequence input.

It outperforms traditional docking tools and shows superior accuracy for various biomolecular interactions^59^. RoseTTAFold All-Atom (RFAA) is another notable generalist method. RFAA is highly advantageous for its ability to accurately model a diverse range of biomolecular assemblies and uniquely offers de novo design of novel small molecule-binding proteins with custom pockets through RFdiffusion All-Atom (RFdiffusionAA), a capability that has been experimentally validated. However, RFAA’s performance in protein–ligand interactions is outperformed by AF3, and RoseTTAFold2NA surpasses it in nucleic acid predictions^60,61^. Even with their advancements, current AI structural prediction tools have limitations. AF3, for example, occasionally faces issues related to chirality, overlapping atoms and a focus on static rather than dynamic structures, and may require numerous predictions to achieve the highest accuracy for complex targets^59^.

Despite these limitations, these AI tools might be crucial for the post-biomarker selection phase. In particular, by providing critical insights into biomarker accessibility and binding dynamics for EV detection assays, they could help bridge the gap between initial biomarker discovery and clinical application.

## Computational evaluation of biomarker candidates

After computational detection and refinement of optimal EV markers as discussed in the previous section, their comprehensive and multifaceted evaluation is important. This evaluation step includes not only experimental validation but also a variety of computational strategies to predict and optimize the performance of candidate EV biomarkers. First, the predictive performance of diagnostic and prognostic biomarkers is assessed using standard classification metrics (for example, area under the receiver operating characteristic curve, accuracy, sensitivity and specificity). For prognostic markers, patient outcomes are evaluated using Kaplan–Meier curves and hazard ratios from Cox models. Beyond statistical significance, the biological context of the selected biomarkers is crucial, integrating biomarkers into known networks to confirm mechanistic plausibility and confirming by cross-validation using independent datasets for generalizability. Finally, potential clinical utility can be assessed by correlating biomarkers with clinical parameters and confirming clinical relevance to existing drugs through drug databases such as ClinicalTrials.gov, Pharos or DrugBank. This systematic evaluation refines candidates, increasing experimental validation success and accelerating clinical translation.

## Perspectives

**Fig. 4:**
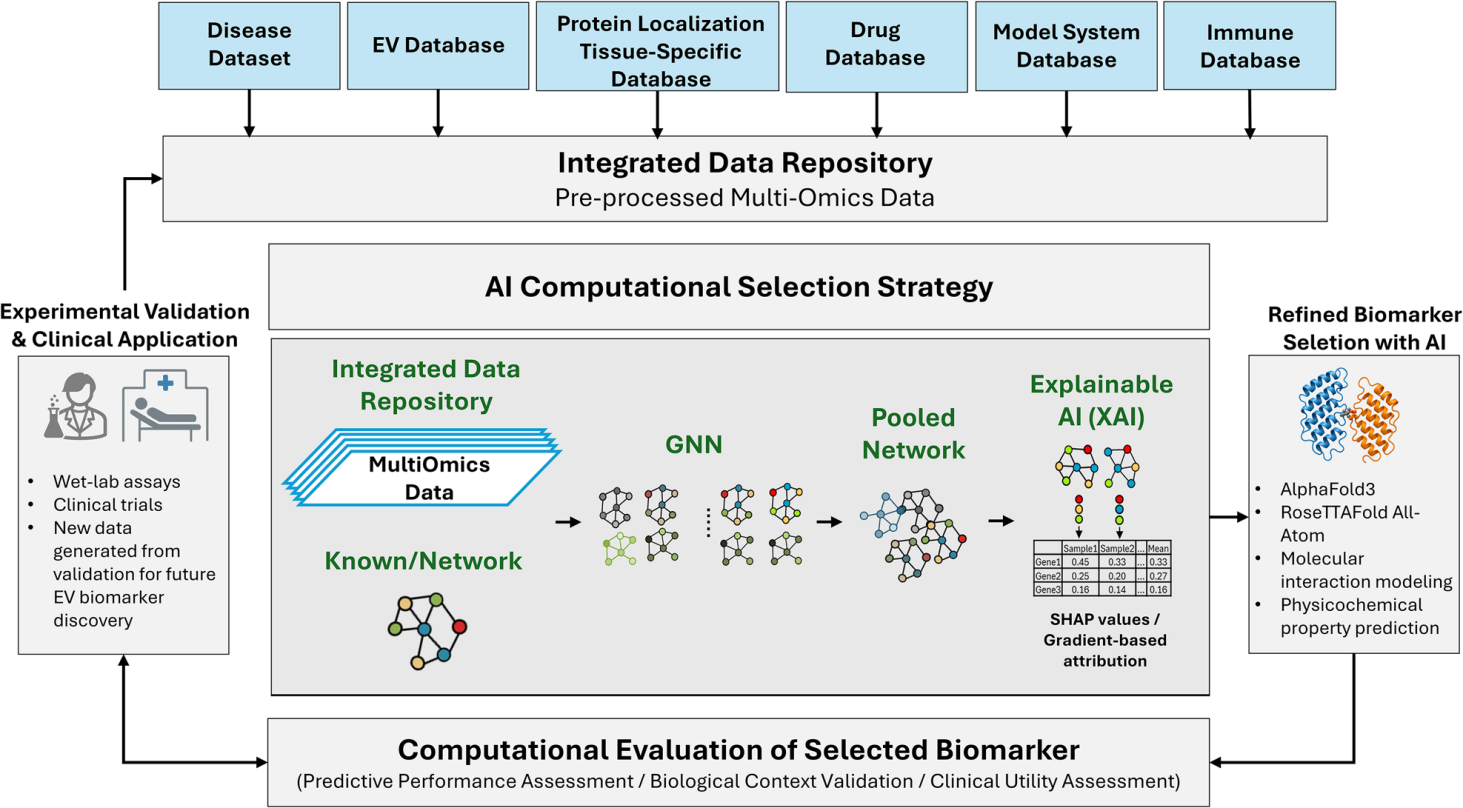
Proposed integrated computational framework. This diagram presents an end-to-end AI pipeline for refined biomarker selection. It integrates GNN for analyzing complex biological networks and Explainable AI (XAI) for interpretable modeling. The framework also utilizes structural tools like AlphaFold3 and RoseTTAFold All-Atom to predict molecular interactions, facilitating clinical validation. GNN,Graph Neural Networks;SHAP,SHapley Additive exPlanations.

To harness the potential resources of AI within the EV biomarker discovery field, it is essential to address several gaps that limit the clinical application of computational results. First, notable data heterogeneity and sparseness exist in the EV research area. Facing these challenges, it is vital to adhere to standardized protocols (for example, MISEV guidelines) for comparable data, as well as to deploy integration methods driven by AI to harmonize disparate sources. Regarding data integration, DL models can provide efficient data integration capabilities. They are capable of directly handling diverse heterogeneous data and automatically learning hierarchical and complex interactions from each type of data. This allows them to effectively detect hidden patterns between multiple omics datasets, revealing synergistic interactions from biomarker signals. However, their lack of interpretability can hinder biological insights. Developing interpretable AI models and incorporating xAI tools, such as SHAP or gradient-based methods, can clarify which EV components (for example, specific proteins or miRNAs) most strongly influence predictions, thereby enhancing both performance and biological understanding. By moving in this direction, we can ensure that AI-driven integration not only delivers high performance in EV biomarker identification but also provides the biological understanding necessary for clinical translation. Second, in parallel, advanced AI tools like AF3 and RFAA can strengthen EV biomarker selection by modeling protein structures and interactions. Incorporating these AI tools into the biomarker selection pipeline will yield valuable insights into candidate proteins’ accessibility, binding and structural stability under specific assay conditions, guiding the selection of optimal EV markers. This process will be a pivotal step in translating computational biomarker findings into high-performance EV detection systems (Fig. 4). Finally, a major barrier in the clinical translation of EV biomarkers is the limited availability of precious clinical samples, which restricts the application of current multi-omic profiling technologies for EVs that may typically demand large input. Advancements in assay platforms capable of detecting low-abundance signals, along with increased support for EV multi-omics consortia and initiatives, could meet wider clinical adoption. This involves creating sophisticated algorithms that effectively reduce noise in both extensive and sparse data.

## Conclusion

AI has transformed biomarker studies by integrating and analyzing complex multi-omics data. Although the use of AI to EV marker discovery is in its early stages, AI has great potential to identify clinically useful markers that cannot be easily detected using conventional methods. Realizing this potential will inevitably require the development of common data practice, interpretable models and seamless integration with experimental validation, ultimately leading to a faster transition of EV biomarkers from discovery to clinical utility and improving precision medicine.
